# Torsion of the Left Layers of the Large Colon in the Horse

**Published:** 1891-12

**Authors:** S. J. J. Harger

**Affiliations:** Professor of Anatomy and Zootechnics in the Veterinary Department of the University of Pennsylvania


### TORSION OF THE LEFT LAYERS OF THE LARGE COLON IN THE HORSE.*

By S. J. J. Harger.

Professor of Anatomy and Zootechnics in the Veterinary Department of the University of Pennsylvania.

At the Congress of Naturalists in Bremen, in 1890, Jelkmann first called attention to the feasibility of, not only diagnosing, in the live animal, a volvulus or torsion of the large colon, but also of treating it by operative means. The importance of this statement is apparent from the frequency of occurrence and the fatality of the alteration. According to Jelkmann's communication, of 192 cases of colic in the horse, seventy suffered from torsion of the folded colon. In twenty-three post-mortems on similar cases, death could in ten be traced to a volvulus of the folded colon. The veterinary statistics of the Prussian army, received in 1886 thirteen, in 1887 twenty-seven, in 1888 thirty-seven, and in 1889 eighty-four death from volvulus. Much credit is due to Jelkmann

* Monatshefte für Practische Thierheilkunde, by Prof. Dr. Möller, of Berlin.

for his careful observation upon this point, and, if his statements are doubted, this is in part excusable from the fact of an insufficient anatomical knowledge by many observers and the indifference with which operative measures have been regarded.

The torsion of the large colon here described, which, according to his remarks, as a rule takes place toward the right, is primarily due to an excessive distension, (especially of the third portion and its pelvic flexure). When the animal, lying down, suddenly rises, the upper layer of the colon, already pushed toward the median line of the abdomen, turns downward, and rotates on its own axis. While this explanation is, in my opinion, significant, it must nevertheless be admitted that the opposite may happen, *i. e.*, torsion toward the left side ; in a case under my observation, the twist was in this direction. The post-mortem records of the local Anatomo-Pathological Institute also confirm it. Sometimes the colon is displaced toward the left, sometimes toward the right. Its relative length often filled with dry contents as well as its extensive mobility in site explains the frequent displacements.

The symptoms of this alteration are not characteristic ; nevertheless, its diagnosis per rectum is not very difficult. Beside the colicky pain, which is moderate but progressively increasing from hour to hour, the diminished peristaltic movements of the intestines, and a small and feeble pulse, the hand introduced into the rectum gives the best information as to the cause of the symptoms. Toward the entrance of the pelvic, the hand can recognize the colon very much distended, which at first can be confounded with the distended bladder. A more critical examination will, however, reveal that it is the large colon. The longitudinal bands of this intestine not only confirm this, but also indicate the direction of the torsion, right or left. In normal conditions, this direction is horizontal, in the direction of the body. In torsion, a change in this direction is distinguished; from before and outwardly, backward and inward, in torsion to the right, and the opposite, when it is toward the left.

Jelkmann states, also, that the great mesentery, whose root from the sub-lumbar region can be outlined, feels more tense, and, in right torsion, instead of being suspended perpendicularly, slants towards the left. Displacing the intestine causes pain. Moreover, the longitudinal bands of the colon are of greater diagnostic value; as soon as they are located, the existence as well as the direction of the torsion are beyond doubt. They need not be confounded with the posterior band on the distended cæcum; the latter can be distinguished by its curvature backward toward the external angle of the right ilium and its termination at the base of the organ.

I have convinced myself of these relations by experiments on the cadaver. The latter being placed in the natural position, the left flank, between the external angle of the ilium, the transverse processes of the lumbar vertebræ and the last rib, was removed. This gave a clear view of the relations of the organs in the postero-abdominal and pelvic cavities. The large colon was then strongly inflated *in situ*, or, after having made the twist, filled with water. The hand per rectum was easily controlled, and could distinguish the relation of the veins, organs, as well as the parts which it touched.

A torsion being now made, the altered conditions could be appreciated and a reposition effected. This proved to me that it is especially necessary to recognize the direction of the colic bands, and, so much more, as the great mesentery is but little displaced. On the contrary, the examination of the form leaves no doubt as to the diagnosis.

Such a displacement of the colon, which does not disappear spontaneously, unless in extremely rare cases from the animal's rolling, always calls for grave prognosis. Hence, the value of the question, is the reduction of the torsion in all cases possible, and, if so, how can it be accomplished? Jelkmann says that, in many instances, success can be obtained. I have convinced myself that this is possible. Great muscular power and eudurance are required, although experience will greatly facilitate it.

An injection of luke-warm water per rectum is first administered to empty the small colon and give room for the hand to work. Jelkmann introduces the left hand into the rectum, directed toward the left flank, and endeavors to push the left (inferior) layers of the large colon, here mixed up with the convolutions of the small colon, forward and toward the middle of the abdominal cavity. Having reached this point, he slowly carries the hand upward, when the colon falls back into its normal position above the hand; while the left lower convolutions of the small colon are being pushed upward, the large colon can fall back into its normal situation. My experiments confirm the success of this operation.

I have had the occasion to reduce a torsion of the colon toward the left side in the following manner: After the small colon was washed out, the right hand followed per rectum the colic bands from in front and inwardly, backward and outward, *i. e.*, backward and to the left. The upper band was then firmly grasped between the palmer surface of the thumb and fingers, and after repeated adductive movements I succeeded finally in drawing it so far to the right as to effect a reduction of the displacement. At this moment, the distension of the pelvic flexure diminished, peristalsis returned, and the small, frequent pulse disappeared; half an hour afterward recovery was insured. As I determined experimentally, the middle band of the inferior surface formed the point of support for the retroversion of a torsion to the left side.

Displacements of the colon cannot be treated after the same and invariable procedure; differences in displacements require variations in the treatment. But, the attention being drawn to the subject, a little experience will enable me to adapt the treatment to individual cases. Perhaps puncture of the distended colon will render the operation more easy.

To explain the preceeding remarks, I may cite the following case : On the 8th of June, I was called to see a heavy draft-horse, which had suffered from colic for twenty hours, during which time no food and but little water had been taken. The examination revealed, moderate but continued uneasiness; the horse laid down and rose frequently, walked to and fro in the stall, and showed all the signs of intestinal impaction, pulse 65, small and weak; respiration 30 per minute and shallow; tympanitis of the posterior part of the abdomen; peristalsis weak, with long pauses, and only on the right side; general sweating, no passage from the bowels, excepting three small balls. Examination per rectum showed the pelvic flexure of the large colon greatly distended with gas, and pushed into the pelvic cavity. On the lateral surface of the colon a strong, tense cord was felt extending from in front, below and inwardly, backward, upward, and outward; a similar cord was outlined on the middle surface of the colon. The bladder contained but little urine.

The hand, per rectum, was directed in the manner described above toward the cord on the lateral surface, and after several laborious attempts, it succeeded in drawing the band toward the median line of the abdomen. This was immediately followed by normal peristalsis, escape of gas, and, very soon a discharge of fæces, the uneasiness ceased, the pulse and the mucous membranes became normal. After an hour recovery was almost complete.

